# Boosting the Oxidative Potential of Polyethylene Glycol‐Based Polymer Electrolyte to 4.36 V by Spatially Restricting Hydroxyl Groups for High‐Voltage Flexible Lithium‐Ion Battery Applications

**DOI:** 10.1002/advs.202100736

**Published:** 2021-06-10

**Authors:** Zhenhan Fang, Yufeng Luo, Haitao Liu, Zixin Hong, Hengcai Wu, Fei Zhao, Peng Liu, Qunqing Li, Shoushan Fan, Wenhui Duan, Jiaping Wang

**Affiliations:** ^1^ Department of Physics and Tsinghua‐Foxconn Nanotechnology Research Center Tsinghua University Beijing 100084 China; ^2^ Laboratory of Computational Physics Institute of Applied Physics and Computational Mathematics Beijing 100088 China; ^3^ Frontier Science Center for Quantum Information Beijing 100084 China; ^4^ State Key Laboratory of Low‐Dimensional Quantum Physics Department of Physics Tsinghua University Beijing 100084 China; ^5^ Institute for Advanced Study Tsinghua University Beijing 100084 China

**Keywords:** gel electrolyte, high‐voltage cathodes, lithium‐ion batteries, oxidation potential, polyethylene glycol

## Abstract

Cross‐linked polyethylene glycol‐based resin (c‐PEGR) is constructed by a ring‐opening reaction of polyethylene glycol diglycidyl ether (PEGDE) with epoxy groups and polyether amine (PEA) with amino groups. By confining the hydroxyl groups with inferior oxidative stability to the c‐PEGR backbone, the oxidation potential of the PEG‐based polymer material with reduced reactivity is boosted to 4.36 V. The c‐PEGR based gel electrolyte shows excellent flexibility, lithium‐ion transport, lithium compatibility, and enhanced oxidation stability, and is successfully applied to a 4.35 V lithium cobaltate (LCO)||lithium (Li) battery system. A quasi‐static linear scanning voltammetry (QS‐LSV) method is proposed for the first time to accurately measure the oxidation potential and electrochemical stability window of materials with low conductivities such as polymers, which possesses the advantages of high accuracy and short test time. This work provides new insights and research techniques for selecting polymer electrolytes for high‐voltage flexible lithium‐ion batteries (LIBs).

## Graphical abstract

A flexible poly(ethylene glycol)‐based resin (c‐PEGR) gel electrolyte with a 3D cross‐linked structure is prepared. The oxidation potential of the c‐PEGR is successfully boosted to 4.36 V by spatially restricting the unstable hydroxyl groups to the backbone, which is successfully applied to a high‐voltage flexible battery system. A quasi‐static linear voltammetry method is proposed to accurately measure the electrochemical stability window of materials with low conductivities.

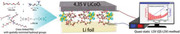

## 1 Introduction

With the gradual advancement of information terminal from the mainframe to wearable devices, electronic equipment is now facing the challenges of miniaturization and flexibilization. As the key components, flexible energy storage devices are used as the power supply in wearable electronics, implantable medical products, and other aspects with broad applications and are expected to promote the revolution of flexible electronics.^[^ ^1^, ^2^, ^3^, ^4^ ^]^ Compared with other mature energy storage devices, lithium‐ion batteries (LIBs) with higher operating voltage and energy density have won the favor of researchers and are considered ideal candidates for flexible energy storage devices.^[^ ^5^ ^]^ Based on traditional LIBs, flexible LIBs are emerged to fulfill even higher requirements of both high energy density and high flexibility. Currently, the following challenges remain in the development of flexible LIBs: (i) Most of the materials used in traditional batteries, whether cylindrical or pouch cells, do not possess the flexibility and are prone to fracture during deformation. (ii) The contact between active materials and other components becomes weak after repeated deformation, resulting in high contact resistance. (iii) The liquid electrolyte has the risk of leakage, causing safety concerns. (iv) It is challenging to achieve high loading of active materials and high energy density while maintaining high flexibility.^[^ ^6^ ^]^ Therefore, the development of novel flexible LIBs and their manufacturing technologies to increase both flexibility and energy density is the foundation for promoting the practical applications of flexible energy storage devices.

Currently, finding suitable electrolyte has become the critical challenge hindering the development of flexible LIBs. The liquid electrolytes used in traditional LIBs have the risk of leakage, especially when the battery undergoes deformation, thus compromising battery safety. In addition, the gas produced during battery assembly and the decomposition of the liquid electrolyte itself may cause the formation of bubbles, resulting in uneven distribution of the electrolyte and the generation of battery inflation. Polymer electrolytes are a promising candidate for use in flexible LIBs due to their excellent flexibility. However, compared to inorganic solid electrolytes and liquid electrolytes, polymer electrolytes suffer from significant drawbacks in low ionic conductivity and narrow electrochemical stability window. Several methods such as grafting,^[^ ^7^, ^8^ ^]^ cross‐linking,^[^ ^9^, ^10^ ^]^ blending,^[^ ^11^, ^12^ ^]^ copolymerization,^[^ ^13^, ^14^ ^]^ organic–inorganic compounding,^[^ ^15^, ^16^ ^]^ and preparation of gel electrolytes,^[^ ^17^, ^18^ ^]^ etc. have been extensively investigated to remarkably improve the ionic conductivity of polymer electrolytes. Still, the low oxidation potential of the polymer electrolytes causes severe decomposition when applied to high‐voltage cathode materials, which hinders the development of flexible LIBs with high output voltage and energy density.

Polyethylene glycol (PEG) is a thermoplastic material with a linear molecular structure and can conduct Li‐ions through complexation/decomplexation of Li‐ions and ethylene oxide (EO) structures, and has been extensively investigated for use as solid electrolytes.^[^ ^19^, ^20^ ^]^ Although PEG has excellent flexibility and processability and can provide sufficient contact with electrodes and conduct Li‐ions, it easily decomposes at the cathode/electrolyte interface at low potentials (3.9 V vs Li/Li^+^), limiting its applications in high‐voltage batteries.^[^ ^21^ ^]^ Many studies have reported attempts to broaden the operating voltage window of PEG‐based polymer electrolytes by organic‐inorganic compounding, copolymerization, etc. However, the intrinsic low oxidation stability of PEGs has not been substantially solved, and it is still challenging to apply PEG electrolyte in high‐voltage batteries. Sun and co‐workers reported that the low oxidation potential of PEG‐based polymer materials was due to the presence of hydroxyl groups at the terminals, which provided insight for understanding the oxidative failure mechanism.^[^ ^22^ ^]^ Based on this finding, polyethylene glycol dimethyl ether (PEGDME) was developed by replacing hydroxyl groups with more stable methyl groups and was successfully applied in batteries with LiNi_0.5_Mn_0.3_Co_0.2_O_2_ (NCM532) cathodes and a cutoff voltage of 4.3 V, achieving higher energy density.

Herein, a cross‐linked polyethylene glycol‐based resin (c‐PEGR) was developed by ring‐opening reaction of poly(ethylene glycol) diglycidyl ether (PEGDE) with epoxy groups and polyether amine (PEA) with amino groups. A gel electrolyte was obtained by swelling the c‐PEGR polymer skeleton in the liquid electrolyte (1 m LiPF_6_ in DMC:FEC), achieving an ionic conductivity (0.7 mS cm^–1^) comparable to that of a liquid electrolyte. In the polymer backbone, the hydroxyl groups were restricted to the cross‐linked structure with limited freedom of movement, which greatly reduced the overall reactivity of the polymer electrolyte and raised its oxidation potential to 4.36 V. LCO||Li cells with the c‐PEGR gel electrolyte exhibited impressive performances at a cutoff voltage up to 4.35 V. In addition, a quasi‐static linear scanning voltammetry (QS‐LSV) method was developed to measure the oxidation potential and electrochemical stability window of polymers with low conductivities. The proposed structure design principles of polymer electrolytes for use in high‐voltage batteries and the method to measure electrochemical window were of substantial guidance in the selection and design of polymer electrolytes for flexible LIBs.

## 2 Results and Discussion

**Figure 1:**
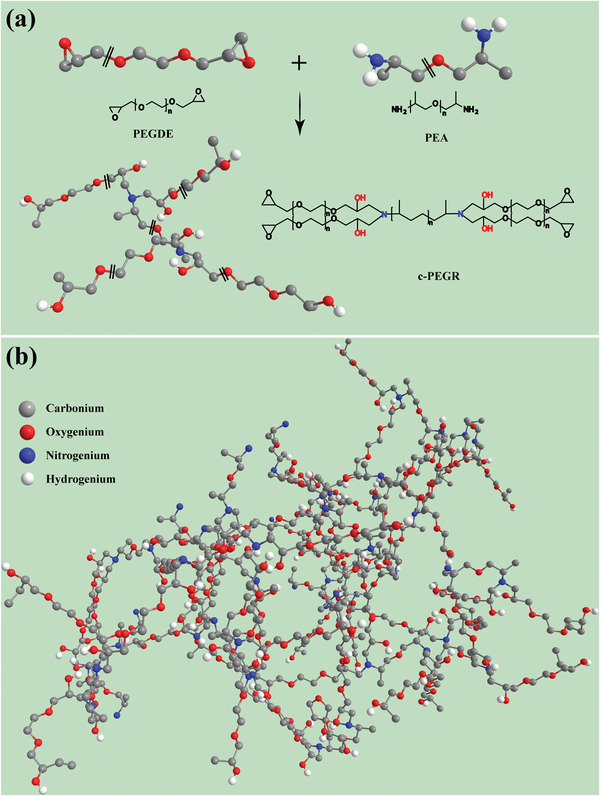
Schematic of a) the synthesis and b) structure of c‐PEGR.

The synthetic schematic of c‐PEGR is shown in **Figure** **1**a, and the synthesis details are elaborated in the experimental section. PEGDE and PEA with either ethylene oxide or propylene oxide structure in the repeating units of the main chain and epoxy and amino‐terminal groups were used to form c‐PEGR by a ring‐opening reaction. Figure 1b illustrates the 3D cross‐linked skeletal structure of c‐PEGR. The hydroxyl groups in c‐PEGR are restricted to the polymer skeleton and show reduced motility, which is distinct from the free‐moving hydroxyl groups in PEG. Therefore, c‐PEGR is much more structurally robust than PEG due to its 3D cross‐linked structure. The prepared c‐PEGR was immersed in a liquid electrolyte (1 m LiPF_6_ in DMC: FEC at a volume ratio of 1:1) for spontaneous swelling to further obtain the c‐PEGR gel. Figure S1a (Supporting Information) presents a schematic of the structure of the c‐PEGR gel, in which the anion, cation, and solvent molecules of the liquid electrolyte are stored in the molecular chains of the c‐PEGR as the structural backbone, and the liquid electrolyte components are the active constituents of the gel electrolyte for Li‐ion conduction. Meanwhile, the hydroxyl groups confined to the polymer skeleton are not altered. The variation of the c‐PEGR's absorbency of liquid electrolyte with time is shown in Figure S1b in the Supporting Information. After 2 h of immersion, the mass of the c‐PEGR gel was saturated, at which point the overall mass of the gel was about 400% of the initial mass of c‐PEGR. The c‐PEGR gel electrolytes used in this work were all produced by swelling c‐PEGR in the liquid electrolyte for 2 h.

**Figure 2:**
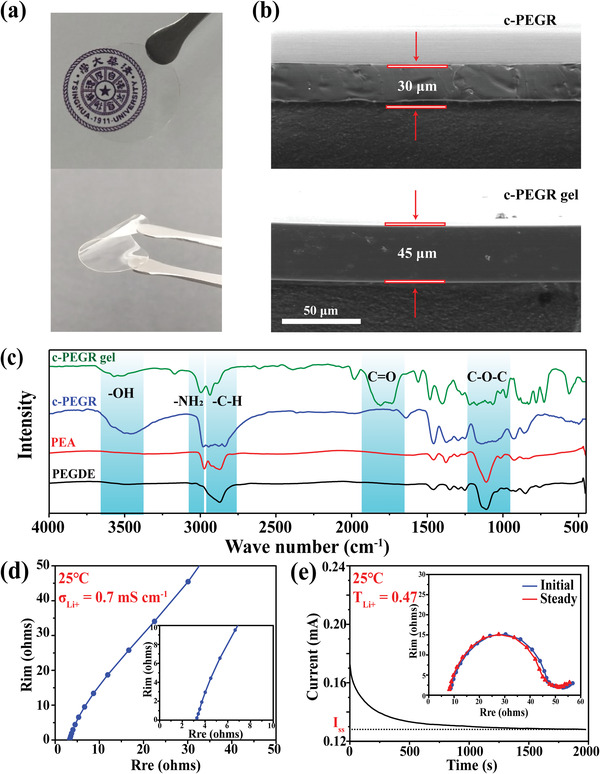
a) Optical photographs of a c‐PEGR gel. b) Cross‐sectional SEM images of c‐PEGR and c‐PEGR gel. c) FTIR spectra of PEA, PEGDE, c‐PEGR, and c‐PEGR gel. d) EIS profile of an Au||c‐PEGR gel|| Au symmetric cell. e) EIS and current profiles of a Li||c‐PEGR gel||Li symmetric cell before and after applying a polarization voltage.

Photograph of a c‐PEGR gel in **Figure** **2**a shows its superior film‐forming properties, transparent appearance, and excellent flexibility. The thickness change of c‐PEGR before and after swelling was obtained by scanning electron microscopy (SEM) characterization of the cross‐section (Figure 2b). c‐PEGR had an original thickness of 30 µm, and the total thickness of the gel electrolyte after swelling for 2 h increased to 45 µm. The reaction process during the c‐PEGR synthesis was investigated by Fourier transform infrared (FTIR) spectroscopy (Figure 2c). Two major peaks near 1100 and 2800 cm^–1^ were detected in both reactants of PEGDE and PEA, corresponding to the stretching vibrations of the ether group (C–O–C) and the carbon‐hydrogen bond (C–H) in the repeating units of the main chain, respectively. Furthermore, PEA exhibited an additional stretching vibration peak near 3000 cm^–1^ due to the presence of the amino group (–NH_2_). The c‐PEGR generated after ring‐opening polymerization of the two reactants additionally exhibited a stretching vibration peak of the hydroxyl group (–OH) near 3500 cm^–1^, which was consistent with the reaction formula in Figure 1a, indicating the formation of the hydroxyl group (–OH) in the c‐PEGR. After immersion of c‐PEGR in a liquid electrolyte, there was a stretching vibration peak at 1800 cm^–1^ in the curve of the c‐PEGR gel due to the presence of a carbonyl group (C═O) in the solvent.

The Li‐ion transportability of the c‐PEGR gel was further tested. A symmetric cell consisting of two gold blocking electrodes and the c‐PEGR gel was assembled for the electrochemical impedance spectrum (EIS) measurement (Figure 2d). The ionic conductivity of the electrolyte was calculated based on the total impedance contributed by the electrolyte and the relevant size of the sample.^[^ ^23^ ^]^ The ionic conductivity of the c‐PEGR gel was 0.7 mS cm^–1^_._ The Li‐ion transfer number of the c‐PEGR gel was determined by the characterization of EIS and steady‐state current as proposed by Evans and co‐workers.^[^ ^24^ ^]^ A Li||c‐PEGR gel||Li symmetric cell was assembled, and the EIS measurement was performed in the initial state and after applying the polarization voltage (Figure 2e). The Li‐ion transfer number of the c‐PEGR gel was calculated to be 0.47. These results demonstrated that both the ionic conductivity and Li‐ion transfer number of the c‐PEGR gel electrolyte are comparable to those of liquid electrolyte at room temperature. The mechanism of Li‐ion conduction in the c‐PEGR gel was different from that in the PEG‐based solid electrolyte. As sufficient liquid electrolyte with greater Li‐ion conductivity was stored in the c‐PEGR gel, the transfer of Li‐ions was no longer dominated by the polymer, which greatly improved the ion conductivity of the c‐PEGR gel.

**Figure 3:**
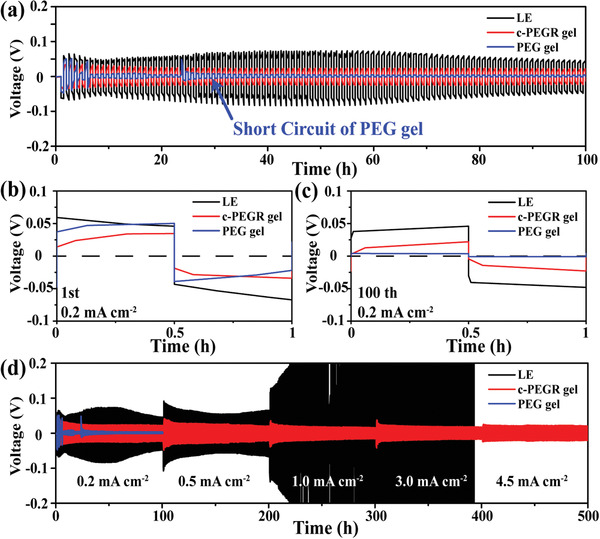
a) Voltage profiles of Li‐symmetric cells assembled with the liquid electrolyte, c‐PEGR gel, and PEG gel at a current density of 0.2 mA cm^–2^ and detailed voltage profiles at the b) 1st and c) 100th cycles. d) Voltage profiles of Li‐symmetric cells at different current densities.

The successful synthesis of c‐PEGR with 3D cross‐linked structure and spatially restricted hydroxyl groups and the following preparation of c‐PEGR gel show great potential for use in Li‐ion batteries. The special structure of the c‐PEGR gel can significantly improve the cycling performance of lithium metal anodes. **Figure** **3**a compares the voltage profiles of Li‐symmetric cells assembled with three different electrolytes: liquid electrolyte, c‐PEGR gel, and PEG gel, at a current density of 0.2 mA cm^–2^. The cell with the c‐PEGR gel exhibited a more stabilized voltage profile with less hysteresis than that with the liquid electrolyte, while the cell with the PEG gel showed an internal short circuit after 30 cycles. The voltage profiles of the Li symmetric cells at the 1st and 100th cycles are shown in Figure 3b,c in detail. For the cell with the c‐PEGR gel, the flat voltage platform (≈25 mV) in both the charged and discharged states was maintained during cycling without a noticeable increase in hysteresis, which was a significant enhancement over that with the liquid electrolyte (≈50 mV voltage platform). These results suggest that the Li‐ion stripping/plating process became much more accessible to the Li metal surfaces with the c‐PEGR gel. For the cell with the PEG gel, the initial overpotential was close to the cell with the liquid electrolyte (50 mV), but due to the lack of structural stability, penetration of Li dendrites and the short circuit occurred, as manifested by a sudden voltage drop during the cycling process. At different current densities, the cell with the c‐PEGR gel exhibited superior cycling stability and lower hysteresis compared to the cells with the liquid electrolyte and the PEG gel (Figure 3d). At current densities higher than 1 mA cm^–2^, the inhomogeneity of the Li metal stripping/plating process became more severe, exacerbating the behavior of dendrite growth, dead Li generation, and the constant consumption of electrolyte by SEI on the Li metal surface. The Li stripping/plating process was more arduous for the cell with liquid electrolyte, manifesting a dramatic increase in overpotential. In contrast, the c‐PEGR skeleton in c‐PEGR gels provided a more robust 3D cross‐linked structure, allowing for improved mechanical strength of the gel electrolyte and more uniform transport of Li‐ions on the lithium metal surface.^[^ ^25^ ^]^ The restricted hydroxyl groups in the c‐PEGR gel had significantly reduced reactivity, which dramatically enhanced the cycling performance of Li metal at high current densities. At ultrahigh current densities of 3 and 4.5 mA cm^–2^, the cell with the c‐PEGR gel maintained an almost identical or even slightly lower overpotentials than those at low current densities due to the gradual activation, demonstrating the great potential of c‐PEGR gel for applications in Li metal batteries.

**Figure 4:**
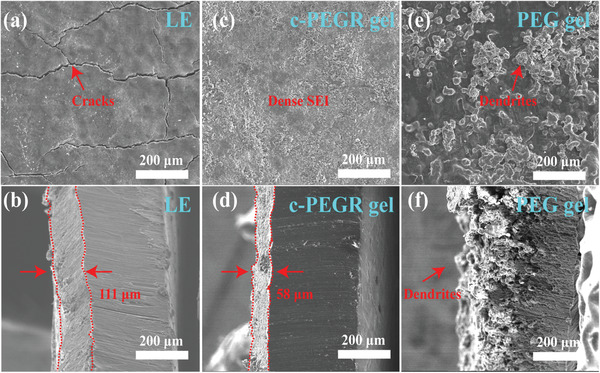
SEM images of the top surface and cross‐sections of Li anodes in cells with the a,b) liquid electrolyte, c,d) c‐PEGR gel, and e,f) PEG gel after cycling.

SEM characterization results showed distinct influences of different electrolytes on Li metal cycling. **Figure** **4**a–f illustrate the top and cross‐sectional morphology of Li foils in symmetric Li cells assembled with the liquid electrolyte, c‐PEGR gel, and PEG gel, respectively, after cycling for 100 h at a current density of 0.2 mA cm^–2^. A 111 µm thick SEI layer with distinct cracks was formed on the surface of the Li anode cycled in the cell with the liquid electrolyte. The presence of cracks suggests that the generated SEI was unstable, and the liquid electrolyte might come into contact with the newly exposed Li through these cracks, leading to further SEI thickening and electrolyte consumption. In contrast, the Li foil cycled in the cell with the c‐PEGR gel was covered with a thinner (58 µm) and denser SEI, which effectively prevented Li dendrite growth and further electrolyte consumption. For the Li anode in the cell with the PEG gel, numerous dendritic particles with inhomogeneous distribution were observed on both the top surface and cross‐section, which revealed the reason for short‐circuiting. According to the above results, the c‐PEGR gel showed an advantage for use as an electrolyte in LIBs compared to the PEG gel. On the one hand, the 3D cross‐linked backbone of c‐PEGR led to the better structural stability of the gel electrolyte. On the other hand, the hydroxyl groups in c‐PEGR were restricted to the cross‐linked skeleton, which impeded them from reaching the electrode surface and obtaining electrons for redox reactions. Therefore, the reactivity of c‐PEGR was greatly reduced compared to that of PEG with freely moving hydroxyl groups.

The better structural stability and reduced reactivity of c‐PEGR owing to the restricted hydroxyl groups in the cross‐linked backbone are beneficial in broadening the electrochemical stability window of PEG‐based polymer materials. Voltammetry was used to measure the redox potential and antioxidant properties of c‐PEGR (Figure S2a,b, Supporting Information). A stainless steel cathode, which was incapable of conducting Li‐ions, was used as the working electrode. Both the reference and counter electrodes were Li foils, between which a c‐PEGR gel or polypropylene (PP) separator and liquid electrolyte were placed for linear sweep voltammetry (LSV) tests at a scan rate of 1 mV s^–1^ in the open‐circuit voltage range up to 6 V. The frontline orbital theory proposed by Goodenough and Kim^[^ ^26^ ^]^ was used to explain the physical mechanism of the oxidation potential measurement, and more specific discussion is provided in Figure S2c–e in the Supporting Information.

**Figure 5:**
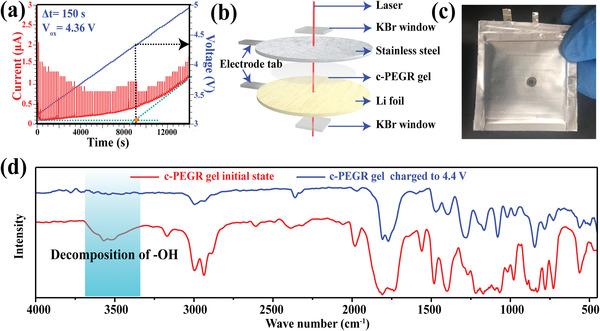
a) Oxidation potential of c‐PEGR gel measured by setting the QS‐LSV at a Δ*t* of 150 s. b) Schematic diagram of the structure and c) photograph of a pouch cell assembled for FTIR measurement. d) FTIR profiles for the c‐PEGR gel at different voltages.

The LSV results surprisingly revealed that the oxidation potential of the c‐PEGR gel was the same as the liquid electrolyte it contained, both at 4.8 V (Figure S2a, Supporting Information). There were two possible situations for this result. One was that the intrinsic oxidation potential of the polymer matrix c‐PEGR in the c‐PEGR gel was higher than the oxidation potential of the liquid electrolyte inside. In this case, the c‐PEGR gel should be able to operate at a maximum cutoff voltage of 4.8 V, which was proven wrong in the subsequent in situ FTIR measurements (**Figure** **5**d). The relevant results demonstrate that the electrolyte decomposition already occurred at a potential below 4.8 V. Another possible situation is that the oxidation potential of the c‐PEGR skeleton could not be tested with LSV. Figure S2b (Supporting Information) shows the oxidation potentials of the c‐PEGR skeleton and c‐PEGR‐LiPF_6_ solid electrolyte. The c‐PEGR skeleton had no oxidation peak in the potential range of 3–8 V, and the oxidation potential of the c‐PEGR‐LiPF_6_ was above 5.6 V corresponding to the decomposition of the lithium salt. These results confirm that the LSV method could not be used to measure the oxidation potential of the c‐PEGR due to the extremely poor electron conduction ability of the polymers. When the chemical potential of the cathode was reduced below the HOMO energy of the polymer, electrons were hardly transported to the cathode to be detected. Therefore, a quasi‐static LSV (QS‐LSV) method was proposed for this situation. As illustrated in Figure S3 (Supporting Information), the QS‐LSV measurement was performed in a way that was not driven by the scan rate (mV s^–1^), but rather kept each voltage value *U* for a duration of Δ*t*, providing sufficient time for the electrons to be diverted to the cathode. At the end of Δ*t*, an additional Δ*U* was added to the previous voltage value, and again, the voltage value *U*+Δ*U* was held for the duration of Δ*t*, and so on. It was the ΔU that determined the deviation of the potential measurement, and the relaxation time Δ*t* guaranteed that the kinetics of electron transport was fully carried out. Figure S3a (Supporting Information) shows the oxidation potential measurement of the c‐PEGR gel using the QS‐LSV method at Δ*t* of 300 s. The red line represents the current and the blue step‐like line represents the voltage. Both the current and voltage varied with the test time. It is apparent that an oxidation peak of c‐PEGR skeleton existed before the oxidation peak of the liquid electrolyte, compared to the same oxidation potentials of the c‐PEGR and the liquid electrolyte obtained from the traditional LSV test (Figure S2a, Supporting Information). These results suggest that in the QS‐LSV method, the electrons generated by oxidation in the polymer can be detected given a duration of Δ*t* that allowed enough time for the electrons to transmit to the cathode. By amplifying the onset position of the c‐PEGR oxidation peak (Figure S3b, Supporting Information), it can be observed that the oxidation potential of c‐PEGR was 4.36 V, which was directly obtained from the voltage value at the corresponding test time. In Figure 5a, a Δ*U* of 0.02 V and a Δ*t* of 150 s were set, and the oxidation potential of the c‐PEGR gel measured by the QS‐LSV method was 4.36 V as well. Keeping the Δ*U* fixed, the test was performed after increasing the Δ*t* (Figure S4, Supporting Information), and the results show that the value of oxidation potential measured by the QS‐LSV method had reached convergence when the Δ*t* lasted longer than 150 s. These results indicated that the electrons involved in the oxidation could fully migrate to the cathode after 150 s and also proved that the QS‐LSV method was self‐consistent.

The QS‐LSV method was essentially based on the traditional LSV method except that the QS‐LSV could provide sufficient migration time for the electrons. Moreover, the QS‐LSV also benefited from the feasibility of controlling both Δ*U* and Δ*t* parameters to regulate the measurement accuracy and duration. For the traditional LSV method, there were also ways to increase the measurement accuracy of the electrochemical window for insulating polymer materials, such as using an extremely slow scan rate for the test. Figure S5 (Supporting Information) demonstrates the oxidation potential profile of the c‐PEGR gel measured by the traditional LSV method at an extremely slow scan rate of 0.01 mV s^–1^, and the test time was as long as 300 000 s in the voltage range of 3–6 V. Still, only the oxidation potential (4.8 V) of the liquid electrolyte in the c‐PEGR gel could be distinguished. At this slow scanning rate, it gave more relaxation time for the electrons to transmit to the cathode. Although it was possible for the oxidation peak of c‐PEGR to appear at lower potentials, the currents were too low to be distinguishable because of the discontinuous shift in voltage and the consequent huge noise of the current signal. The time required to complete the LSV test at a scanning rate of 0.01 mV s^−1^ was much longer than that required for the QS‐LSV test (in the voltage range of 3–6 V with Δ*U* of 0.02 V and Δ*t* of 150 s), but accurate results of oxidation potential could not be obtained by the LSV test. Instead, the oxidation potential of the c‐PEGR gel was accurately measured by the QS‐LSV method at a much shorter time. Therefore, the QS‐LSV method has the advantages of high accuracy and short test time compared with the traditional LSV method when measuring the electrochemical stability windows of materials with low electronic conductivities such as polymers.

FTIR spectroscopy measurements of the c‐PEGR gel validated the accuracy of the results of the QS‐LSV measurement. Schematic and optical photographs of the FTIR testing apparatus are presented in Figure 5b,c. A perforation was drilled in the aluminum‐plastic film of the pouch cell, and potassium bromide (KBr) windows were mounted inside of the aperture. Epoxy glue was used to seal the KBr windows with the aluminum‐plastic film. Holes with 0.1 mm diameter were drilled in the stainless steel and Li foil to allow the IR beam to transmit. The red and blue curves in Figure 5d correspond to the initial state of the c‐PEGR gel and the state charged to 4.4 V, respectively. When the potential reached 4.4 V, the peak at 3500 cm^–1^ disappeared, corresponding to the decomposition of hydroxyl in the c‐PEGR, which agrees well with the oxidation potential of 4.36 V measured by the QS‐LSV method.

**Figure 6:**
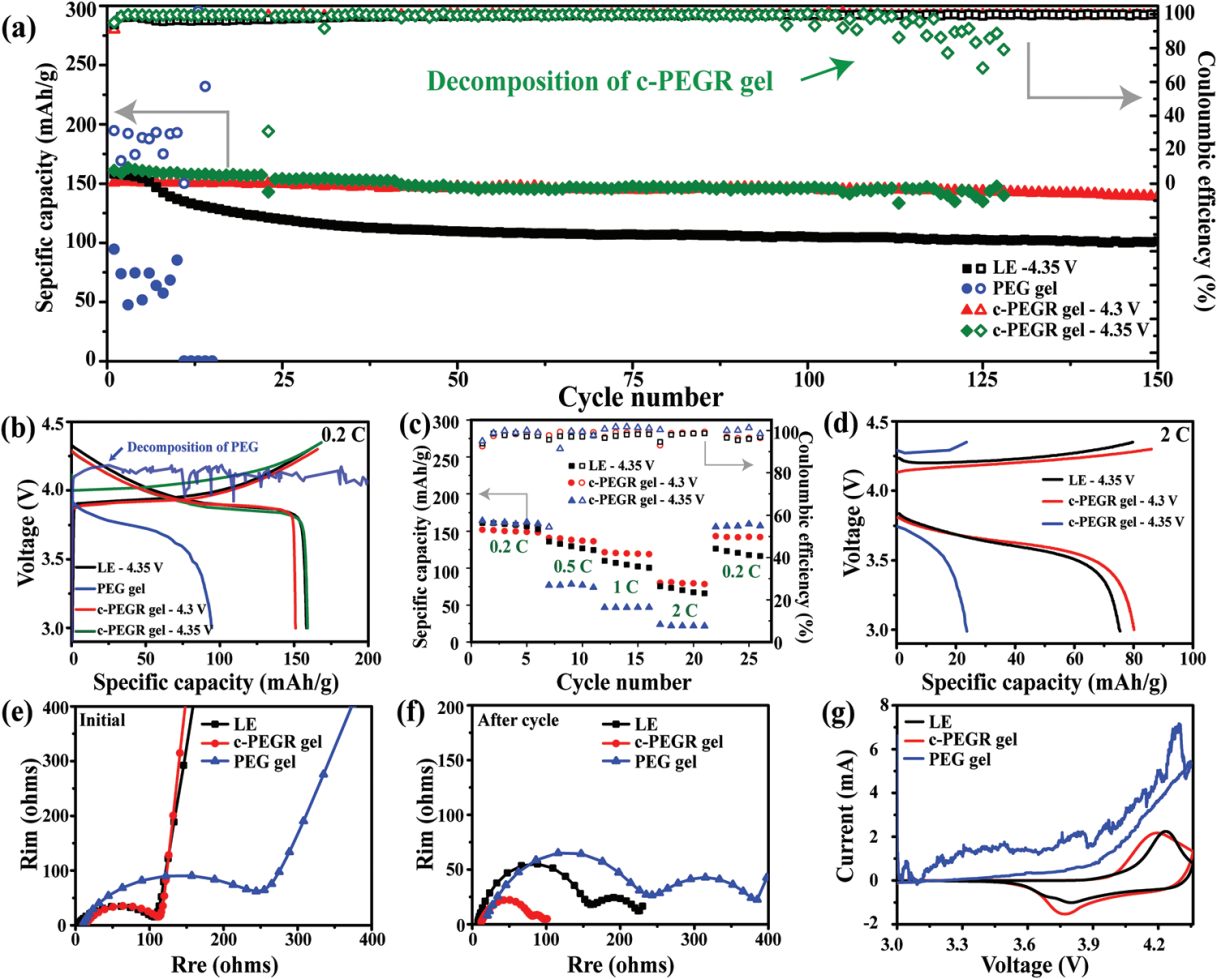
a) Cycling performance at 0.2 C, b) voltage profiles, and c) rate performance of LCO||Li cells with the liquid electrolyte, c‐PEGR gel, and PEG gel. d) Voltage profiles of LCO||Li cells with the liquid electrolyte and c‐PEGR gel at 2 C. EIS curves in the e) initial state and f) after cycling and g) CV profiles of LCO||Li cells.

Based on the above measurements, it was verified that the oxidation potential of c‐PEGR was successfully raised to 4.36 V, enabling its use in LCO||Li cells at higher working voltages. **Figure** **6** shows the cycling performance of LCO||Li cells with the liquid electrolyte, c‐PEGR gel, and PEG gel at 0.2 C. When the cutoff voltage was increased to 4.35 V, the cell with the c‐PEGR gel was still able to operate, exhibiting an initial capacity of 159.1 mAh g^–1^. After 100 cycles, the capacity was retained at 146.3 mAh g^–1^ with 91.95% capacity retention and 99.92% average coulombic efficiency. In contrast, the cell with the PEG gel exhibited unstable capacity and low coulombic efficiency at high operating voltages due to its inferior oxidative stability and failed after ten cycles. These electrochemical performance results confirm the role of the restricted hydroxyl groups in c‐PEGR in improving its oxidative stability. In addition, the LCO||Li cell with the c‐PEGR gel exhibited better cycling stability and higher coulombic efficiency compared to the cell with the liquid electrolyte, as the cross‐linked structure of c‐PEGR contributed to the enhanced cycling stability of lithium anode (Figure 4). However, the cell assembled with the c‐PEGR gel experienced a decline in coulombic efficiency after 110 cycles at a cutoff voltage of 4.35 V. Since the cutoff voltage of 4.35 V was extremely close to the oxidation potential of c‐PEGR, the polarization of the cell gradually increased during cycling, and there was a risk of c‐PEGR decomposition under greater polarization. Therefore, the cutoff voltage was set at 4.3 V to achieve a more stable cycling performance. The cell with the c‐PEGR gel exhibited an initial discharge specific capacity of 149.9 mAh g^–1^ and maintained 138.4 mAh g^–1^ after 150 cycles, showing a high capacity retention of 92.33% and average coulombic efficiency of 99.95%.

Voltage profiles of the LCO||Li cells at a cutoff voltage of 4.3 V are shown in Figure 6b. The cell with the c‐PEGR gel showed almost the same curve as that with the liquid electrolyte, indicating the superior Li‐ion conduction capability of the gel electrolyte. Moreover, by slightly lowering the cutoff voltage from 4.35 to 4.3 V, the cell with the c‐PEGR gel presented a smaller polarization. For the cell with the PEG gel, in contrast, a long, disordered platform was displayed near 4.1 V, corresponding to the capacity contributed by the decomposition of PEG. Since this part of capacity was irreversible and the PEG decomposition products covering the electrode surface led to increased polarization, the cell with the PEG gel exhibited a discharge specific capacity of only 94.5 mAh g^–1^ and a low coulombic efficiency of 31.22%. Figure 6c shows the rate performance of the cells with the liquid electrolyte and c‐PEGR gel at cutoff voltages of 4.3 and 4.35 V. At a safer cutoff voltage of 4.3 V, the cell with the c‐PEGR gel demonstrated the best performance at different rates. Specifically, the specific capacities were 152, 140.9, 121.5, and 80.1 mAh g^–1^ at 0.2, 0.5, 1, and 2 C, respectively, and remained at 143.3 mAh g^–1^ when the current density was restored to 0.2 C. The voltage profiles at 2 C (Figure 6d) show more clearly the performance of the cells assembled with the c‐PEGR gel at different cutoff voltages. The blue line indicates the voltage–capacity curve of the cell with the c‐PEGR gel at a cutoff voltage of 4.35 V. Because the cutoff voltage was extremely close to the oxidative decomposition potential of c‐PEGR, the larger polarization induced by the high current density at a 2 C led to the performance degradation. The red line represents the voltage profile of the cell with c‐PEGR gel at a cutoff voltage of 4.3 V. By adjusting the cutoff voltage from 4.35 to 4.3 V, the oxidative decomposition of c‐PEGR due to polarization was greatly reduced. Moreover, the specific capacity was higher than the cell assembled with the liquid electrolyte at a cutoff voltage of 4.35 V. These results demonstrate that compared to the liquid electrolyte and PEG gel, the c‐PEGR gel with restricted hydroxyl groups in the cross‐linked backbone possessed higher oxidation stability and contributed to the significantly enhanced performance of LCO||Li cells.

EIS curves and cyclic voltammetry (CV) results of LCO||Li cells with the liquid electrolyte, c‐PEGR gel, and PEG gel are shown in Figure 6e–g. The c‐PEGR gel possessed exceptional structural stability, Li‐ion transportability, and excellent flexibility to make sufficient contact with the electrode. Therefore, there was no significant difference in charge transfer resistance between the cells with the liquid electrolyte (102.3 Ω) and the c‐PEGR gel (101.9 Ω) at the initial stage. The PEG gel had less structural stability, and the cell with the PEG gel exhibited a higher charge transfer impedance of 265.7 Ω at the initial state. After cycling at 0.2 C, the generated cathode electrolyte interface (CEI) on the cathode surface and the newly formed SEI on the anode were represented as two semicircles in the high‐frequency and low‐frequency regions in the EIS curves. After cycling, the cell with the c‐PEGR gel exhibited the best Li‐ion transfer capability. The charge transfer resistances at the cathode and anode interfaces were 71.2 and 25.5 Ω, respectively, much lower than those in the cells with the liquid electrolyte (156.5 and 81.5 Ω) and the PEG gel (239.9 and 183.4 Ω). These results suggest that the cell with the c‐PEGR gel possessed the thinnest passivation layer and easiest Li‐ion transferability, resulting in the best cycling stability. Figure 6g reveals the CV measurements of LCO||Li cells with the liquid electrolyte, c‐PEGR gel, and PEG gel during the first cycle. The voltage range was set to 3.0–4.36 V. The oxidative decomposition of the PEG gel occurred at the first charge, consistent with the voltage profile in Figure 6b. A reduction peak and an oxidation peak were observed in the cells with the liquid electrolyte and c‐PEGR gel, corresponding to the lithiation/delithiation of Li‐ions in LCO during the cycling process. The long‐term cycling performance of the LCO||Li coin cell with the c‐PEGR gel is demonstrated in Figure S6 in the Supporting Information. It had an initial discharge specific capacity of 148.5 mAh g^–1^ at a rate of 0.1 C and still retained a discharge specific capacity of 95.7 mAh g^–1^ after 300 cycles with a capacity retention of 64.44% and an average coulombic efficiency of 99.85%.

**Figure 7:**
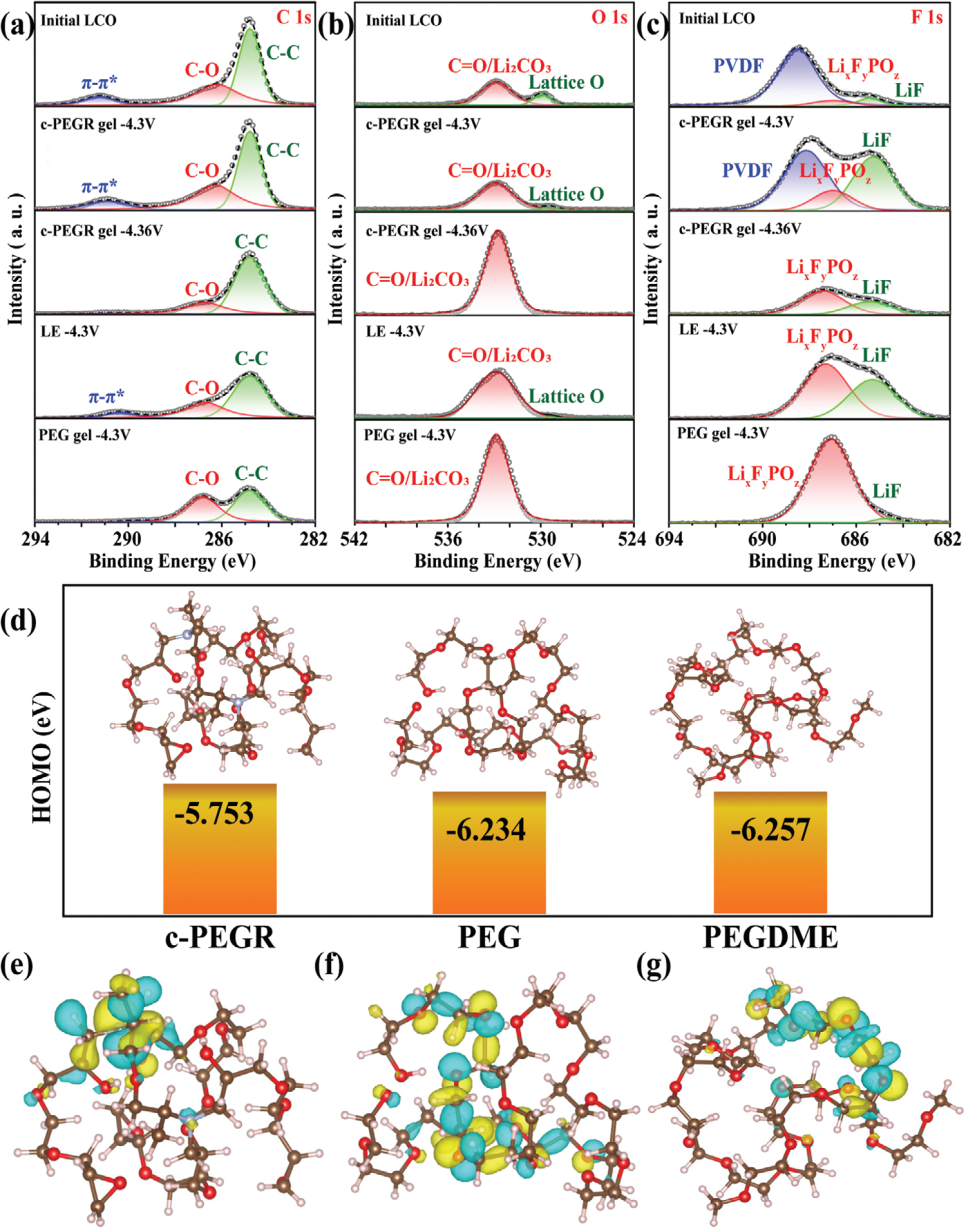
XPS spectra of a) C 1s, b) O 1s, and c) F 1s of the LCO cathode disassembled from LCO||Li cells with the liquid electrolyte, c‐PEGR gel, and PEG gel after cycling. d) The HOMO energy levels (The H, C, N, and O atoms were represented with the pink, brown, light blue, and red balls, respectively.) and e–g) the HOMO distribution visualization of c‐PEGR, PEG, and PEGDME. The isosurface value was set to 0.03 a.u.

To further investigate the cathode/electrolyte interface, the evolution of CEI during cycling was studied by X‐ray photoelectron spectroscopy (XPS). **Figure** **7**a shows the C1s spectra collected from the initial LCO, the LCO after cycling at a cutoff voltage of 4.3 V in the cells with the liquid electrolyte, c‐PEGR gel, and PEG gel, respectively, and the LCO after cycling at a further increased cutoff voltage of 4.36 V in the cell with the c‐PEGR gel. For the C 1s spectra, three peaks occurred at 284.8, 286.2, and 291.2 eV, corresponding to the C–C, C–O, and *π*–π* bonds contained in the initial LCO cathode, respectively.^[^ ^27^ ^]^ The *π*–π* bond was ascribed to the contribution of the conducting agent (super‐P) contained in the cathode. Considering the limited analytical depth of XPS, this peak with a binding energy of 291.2 eV was used as an indicator of CEI thickness. For the cell with the c‐PEGR gel, the intensity and proportion of the peaks in LCO after cycling at a cutoff voltage of 4.3 V were almost the same as those at the initial state, suggesting that the c‐PEGR gel did not undergo oxidative decomposition at a cutoff voltage of 4.3 V. Only an extremely thin CEI passivation layer was generated on the LCO surface, which was consistent with the stable cycling performance of the cell. When the cutoff voltage increased to 4.36 V, a much thicker CEI formed on the LCO surface due to the oxidative decomposition of c‐PEGR, and the *π*–π* peak at 291.2 eV disappeared. For the cell with the liquid electrolyte, the relative intensities of the super‐P peak and the C–C peak contributed by the binder poly(vinyl difluoride) (PVDF) decreased after cycling at a cutoff voltage of 4.3 V, indicating that a CEI of appropriate thickness was generated on the LCO surface. For the cell with the PEG gel, an even thicker CEI formed due to the oxidative decomposition of PEG, and the super‐P peak disappeared after cycling at a cutoff voltage of 4.3 V. The C1s spectra results suggest the formation of the thinnest CEI passivation layer on LCO surface in the cell with the c‐PEGR gel due to its structural and oxidative stability. Spectra of both O 1s and F 1s show similar results. For the O 1s spectra, two peaks of 532.9 and 529.9 eV corresponded to O in C═O/Li_2_CO_3_ and LCO lattices, respectively.^[^ ^28^ ^]^ After cycling at a cutoff voltage of 4.3 V, the peak of lattice O remained for the cell with the c‐PEGR gel and almost disappeared for the cells with the liquid electrolyte and PEG gel, suggesting the formation of the thinnest CEI passivation layer on the LCO surface in the cell with the c‐PEGR gel. When the cutoff voltage increased to 4.36 V, the peak of lattice O disappeared for the cell with the c‐PEGR because of the thicker CEI formed at higher voltages. The F 1s spectra mainly contained three peaks: LiF (685.3 eV), Li*_x_*F*_y_*PO*_z_* (687 eV), and PVDF (687.9 eV).^[^ ^29^ ^]^ LiF and Li*_x_*F*_y_*PO_z_ were the main F‐containing components of CEI in LiPF_6_‐based electrolytes, leading to severe capacity degradation during cycling, especially at high voltages. The PVDF peak only showed in the LCO sample after cycling in the c‐PEGR gel at a cutoff voltage of 4.3 V, indicating that the thickness of the CEI was the thinnest among all samples. The XPS spectra demonstrate that a more stable cathode/electrolyte interface was achieved at a high cutoff voltage in the cell with the c‐PEGR gel with a cross‐linked backbone structure compared to those with the liquid electrolyte and the PEG gel.

Theoretical calculations were used to demonstrate how the restricted hydroxyl groups in c‐PEGR could decrease the polymer reactivity and broaden the electrochemical window. A small prototype molecule was created to represent the synthesized c‐PEGR, and for comparison, a PEG molecule of similar size was also constructed. A PEGDME molecule that was able to raise the oxidation potential to 4.3 V according to the literature was also compared.^[^ ^22^ ^]^ Figure 7d shows the HOMO energy levels of these polymers. Surprisingly, the HOMO of c‐PEGR (−5.753 eV) was even higher than those of PEG (−6.234 eV) and PEGDME (−6.257 eV), which was contradictory to the experimental result that c‐PEGR had the highest oxidative stability. Upon further visualization of the HOMO isosurfaces of the polymers, the mechanism of the enhancement of oxidative stability was proposed. Figure 7e–g presents the spatial distribution of HOMO for c‐PEGR, PEG, and PEGDME. The yellow and blue regions represent the positive and negative phases of the electron wave function, respectively, and the size of the region indicates the value of the electron wave function. The electrons occupied at the HOMO have the highest energy, and these atoms with a larger contribution of HOMO tend to be the preferential region for the electrophilic attack according to the frontier orbital theory. Since the PEGDME molecule no longer contained hydroxyl groups that were easily oxidized, its HOMO was predominantly distributed in the EO structure of the main chain. The oxidation potential of EO was higher than 4.3 V;^[^ ^22^ ^]^ thus, the oxidation potential of PEGDME was enhanced. Both c‐PEGR and PEG contained hydroxyl groups, but the hydroxyl groups in c‐PEGR were confined to the cross‐linked skeleton, and their reactivity was greatly reduced. The HOMO of c‐PEGR was mainly localized around only a few atoms, while the HOMO of PEG and PEGDME was more delocalized. Therefore, the HOMO orbital of c‐PEGR had a smaller overlap with the empty orbital of the electrophilic reagent (cathode) and was less susceptible to the electrophilic attack at the electrode surface. Thus, the oxidative stability of the c‐PEGR was significantly enhanced. The strategy of spatially restricting groups with poor redox stability is of great practical value for broadening the electrochemical window of polymer materials. Solid‐state nuclear magnetic resonance hydrogen (^1^H‐SSNMR) spectroscopy was performed to characterize the activity of the hydroxyl groups in c‐PEGR. Since the active hydrogen in the hydroxyl group tends to be replaced by deuterium, which cannot be displayed on the ^1^H‐SSNMR spectra, the activity of the hydroxyl groups can be determined by comparing the peak intensities of the hydroxyl groups before and after the deuterium exchange (D‐exchange). The black and red dashed lines in Figure S7 (Supporting Information) represent the intensities of c‐PEGR before and after the D‐exchange, respectively. The peaks with chemical shifts at 4.30, 4.07, and 1.85 ppm correspond to the hydrogen atoms in the EO group, attached to the amine atom, and in the hydroxyl group, respectively. The spectra of c‐PEGR did not change significantly from the initial state after sufficient D‐exchange experiments, and especially the peak intensity of the hydroxyl group remained almost the same before and after the D‐exchange. These results suggest that the hydroxyl groups in c‐PEGR were exchanged with deuterium at a low degree and the activity of the hydroxyl groups was low, which further verified that the spatial restriction of the hydroxyl groups in c‐PEGR dramatically reduced their activity and thus improved the oxidation stability of c‐PEGR.

**Figure 8:**
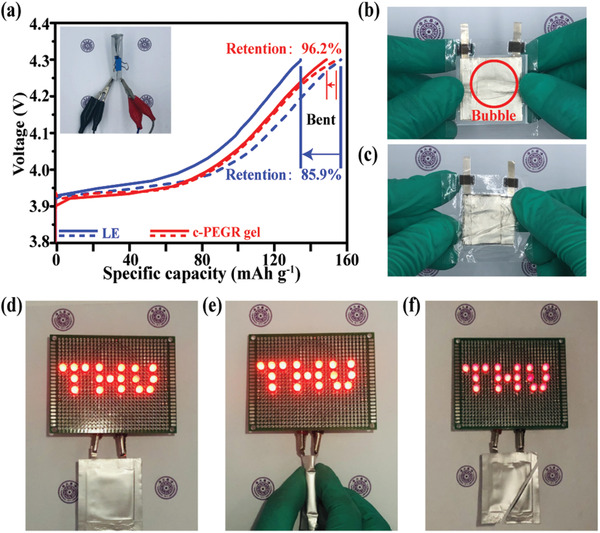
a) Charge voltage–capacity curves of pouch cells with the liquid electrolyte and c‐PEGR gel before and after bending. Optical photographs of pouch cells with the b) liquid electrolyte and c) c‐PEGR gel after bending. Optical photographs of a pouch cell with the c‐PEGR gel lighting up an LED array at different conditions: d) flat; e) bent; f) cut.

The c‐PEGR gel not only had superior Li‐ion transportability and could operate at high voltages but also possessed a unique advantage for use as an electrolyte in flexible batteries due to its excellent flexibility. The blue and red dashed lines in **Figure** **8**a represent the voltage–capacity curves of flexible pouch cells with the liquid electrolyte and c‐PEGR gel during the first charge process at a rate of 0.1C, and similar initial charge capacities of 156.7 and 154.7 mAh g^–1^ were achieved. Once the cells were bent, as shown in Figure 8a inset, significant degradation of the specific charge capacity occurred for the cell with the liquid electrolyte. The blue and red solid lines in Figure 8a indicate the charging curves of the pouch cells with the liquid electrolyte and c‐PEGR gel after bending. For the pouch cell with the liquid electrolyte, the capacity retention was only 85.9%, while the cell with the c‐PEGR gel exhibited high capacity retention of 96.2%. The internal changes in the cells during bending are visible in Figure 8b,c. For the cell with the liquid electrolyte, when the pouch cell was bent, electrolyte flow occurred, resulting in an uneven electrolyte distribution inside the cell. The presence of bubbles is observed in Figure 8b, which caused the risk of leakage and capacity degradation. In the cell with the c‐PEGR gel, the electrolyte was well stored in the gel due to the presence of the c‐PEGR skeleton, which maintained sufficient contact with the electrode even when the cell was bent and also reduced the risk of leakage. Figure 8d shows an optical photo of a pouch cell with c‐PEGR gel lighting up a light‐emitting diode (LED) array, which remained lit when the battery was bent (Figure 8e). Even after a corner of the cell was cut off, the electrodes and electrolyte remained in constant contact, and the cell still functioned well and did not leak (Figure 8f). These experiments verified that the c‐PEGR gel, by storing liquid electrolyte inside the c‐PEGR, can be an excellent electrolyte material for flexible batteries by ensuring excellent Li‐ion transport while keeping tight contact with the electrode when the battery was subjected to mechanical deformation.

## 3 Conclusion

c‐PEGR with a cross‐linked structure was fabricated by ring‐opening polymerization of PEGDE and PEA. The EO/PO structure in the main chain implied excellent compatibility with the Li metal anode. Meanwhile, the hydroxyl groups were successfully confined to the polymer skeleton. Experimental results and theoretical calculations demonstrate that the oxidative stability of the PEG‐based polymers could be effectively enhanced by spatially limiting the motility of hydroxyl groups, mainly due to the significantly reduced possibility of charge transfer by the hydroxyl groups anchored inside the nonconductive polymer. Furthermore, a QS‐LSV method was proposed to accurately measure the oxidation and reduction potentials of materials with low electrical conductivities such as polymers. Specifically, a relaxation time was maintained at each testing potential to permit adequate time for electron transfer to the cathode. The QS‐LSV test had the advantages of high accuracy, short test time, and applicability to materials with low conductivities. The oxidation potential of c‐PEGR was measured to be 4.36 V by the QS‐LSV method. The c‐PEGR gel was successfully applied in LCO||Li batteries with a cutoff voltage of 4.35 V, dramatically upgrading the applicability of PEG‐based polymers in high‐voltage batteries. Moreover, due to its excellent flexibility, c‐PEGR became a promising candidate as an electrolyte material for high‐voltage flexible LIBs.

## 4 Experimental Section

### Preparation of the c‐PEGR Gel Electrolyte

PEGDE (Macklin, average molecular weight ≈ 400) and PEA (Aladin, average molecular weight ≈ 2000) were formulated in equal equivalents according to respective epoxy and amine values. A precursor with a volume ratio of PEGDE: PEA = 7:20 was magnetically stirred at 55 °C and evenly coated on a poly tetra fluoroethylene (PTFE) substrate. Finally, the temperature was raised to 85 °C and maintained for 48 h to obtain c‐PEGR. The prepared c‐PEGR was further immersed in 1 m LiPF_6_ solution in DMC:FEC at a volume ratio of 1:1 to obtain the c‐PEGR gel electrolyte.

### Preparation of the PEG Gel Electrolyte

2 g PEG (Aladin, average molecular weight ≈ 100 000) was mixed in 10 mL of acetonitrile (Macklin) and stirred at 65 °C for 12 h until the PEG was completely dissolved. The solution was evenly coated on a PTFE substrate, and the PEG film was obtained after the acetonitrile evaporated. When the coin cell was assembled, a PEG film with a diameter of 19 mm was cut as a separator, and 60 µL of liquid electrolyte (1 m LiPF_6_ in DMC: FEC = 1:1 vol%) was injected so that the PEG film spontaneously absorbed the electrolyte to form a PEG gel.

### Absorbance Measurement of c‐PEGR

The prepared c‐PEGR was weighed to obtain the initial mass (*M*_0_). After immersion in the liquid electrolyte for a specified time, the surface residue gel was removed, and then the total mass of the c‐PEGR gel (*M*′) was measured. The absorbance was defined using the following equation

(1) $$\mathrm{Absorbency}=\frac{M^{\prime }-M_{0}}{M_{0}}\times 100\%$$

### Ionic Conductivity Measurement of the c‐PEGR Gel

Gold blocking electrodes were used as the working, reference, and counter electrodes for coin cell assembly, and the c‐PEGR gel was used as the electrolyte. EIS tests were conducted on an electrochemical workstation with a frequency range of 1 MHz to 1 mHz and a perturbation amplitude of 10 mV. The ionic conductivity (*σ*) of the c‐PEGR gel was calculated as

(2) $$\sigma =\frac{d}{R_{b}\times S}$$

*d* and *S* were the thickness of the c‐PEGR gel electrolyte and the effective area of the electrode, respectively. *R*_b_ represented the bulk resistance as depicted by the intercept of the extended semicircle on the real axis in the EIS plot.

### Li‐Ion Transference Number Measurement of c‐PEGR Gel

The Li‐ion transference number was evaluated using EIS and steady‐state current tests. A two‐electrode system was used for testing. The c‐PEGR gel electrolyte was sandwiched between two Li electrodes and assembled into a Li||c‐PEGR gel||Li symmetric cell. The bulk resistance (*R*_b_) of the c‐PEGR gel electrolyte and the initial interface resistance *R*_0_ with the Li electrode were obtained by the EIS tests at 1 MHz to 0.1 Hz. A small polarization voltage (10 mV) was then continuously applied to the cell, and the polarization voltage was withdrawn when the polarization current decayed to a stable value *I*ss, after which the EIS measurement was performed on the cell again. The new bulk resistance (*R*_b_′) of the c‐PEGR gel and the final interface resistance *R*_f_ with the Li electrode were derived. The Li‐ion transference number was calculated according to the following equation

(3) $$T_{Li^{+}}=\frac{I_{\mathrm{ss}}}{I_{0}}\left(\frac{\Delta V-I_{0}R_{0}}{\Delta V-I_{\mathrm{ss}}R_{f}}\right)$$

where Δ*V* was the polarization voltage and the initial polarization current *I*_0_ was calculated using the following equation

(4) $$I_{0}=\frac{\Delta V}{R_{b}+R_{0}}$$

### Theoretical Study of the Electronic Properties of Polymers

A conformer‐rotamer ensemble sampling tool^[^ ^30^, ^31^ ^]^ based on the semiempirical tight binding method GFN2‐xTB^[^ ^32^, ^33^, ^34^ ^]^ was adopted for conformational searching of such a long‐chain polymer molecule. Then the lowest energy conformer was chosen for subsequent geometry optimization and electronic structure analysis with the density functional theory (DFT) by the ORCA package.^[^ ^35^ ^]^ The Becke three‐parameter Lee–Yang–Parr hybrid functional (denoted as B3LYP) was employed,^[^ ^36^ ^]^ in combination with the Grimme's D3 dispersion correction with Becke‐Johnson damping scheme.^[^ ^37^, ^38^ ^]^ The def2‐SV(P) and def2‐TZVPP basis sets^[^ ^39^ ^]^ were used for the geometry optimization and the final electronic structure calculation, respectively. The visualization of the geometrical structure and molecular orbital was fulfilled by the VESTA program.^[^ ^40^ ^]^

### Assembly of Coin and Pouch Cells

The assembly of cells was performed in an argon glove box (M. Braun Inert Gas Systems Co., Ltd., Germany). Coin‐type (CR 2016) half‐cells were assembled with LCO as the working electrode, the prepared c‐PEGR gel as the electrolyte, and Li foil (China Energy Lithium CO., LTD.) as both the counter and the reference electrode. The LCO electrode was prepared by dispersing LCO powder (10 µm, Reshine, China), conductive super P, and PVDF at a weight ratio of 8:1:1 in *N*‐methyl‐2‐pyrrolidine solution. Then the slurry was coated onto an aluminum foil via the commercially used slurry‐coating method and dried at 80 ℃. Eventually, the dried LCO sheet was cut into 10 mm diameter discs with an LCO loading of 7 mg cm^–2^. For the assembly of pouch cells, a 3 cm × 3.4 cm LCO cathode was spot‐welded with a 0.4 cm wide aluminum lead for use as the cathode. The anode was made by cold pressing a 3 cm × 3.4 cm Li foil with a 0.4 cm wide nickel lead. The c‐PEGR gel with a size of 4 cm × 4 cm was wedged between cathode and anode before encapsulation of the pouch cell by an aluminum‐plastic film.

### Electrochemical Measurements

For LCO||Li batteries, electrochemical measurements were performed using a Land CT2001 automated battery tester for coin cells, and a Neware battery tester for pouch cells at the specified voltage of 3.0–4.3 V. CV and EIS measurements were conducted on the PARSAT2273 (AMETEK) and Vertex. C (IVIUM) electrochemical workstation with a voltage range of 3.0–4.36 V and a scan rate of 0.1 mV s^–1^. EIS characterization was performed with a small perturbation voltage of 2 mV and a frequency range of 100 kHz to 100 mHz. All electrochemical tests were carried out at room temperature and in an ambient atmosphere.

### Morphology and Structural Characterization

SEM (Sirion 200, FEI, USA) was used to characterize the morphology and structure of the c‐PEGR and c‐PEGR gel. Vertex 80/80V Fourier Transform Infrared Spectrometer from Bruker Optics was used to perform the FTIR measurements. XPS data were collected from a PHI Quantera II (Ulvac‐Phi Inc) at room temperature. ^1^H‐SSNMR spectra were measured using a solid‐state NMR instrument (JNM‐ECZ600R). The resonance frequency was 600 MHz, the tube diameter was 3.2 mm, and the magic angle spinning frequency was 12 kHz. The D‐exchange fully occurred by mixing 0.1 mL of solid material (c‐PEGR) with 0.5 mL of deuterium oxide (D_2_O) and maintaining the mixture at 60 ℃ for 12 h. Afterwards, the water was removed in a vacuum oven at 100 ℃.

## Conflict of Interest

The authors declare no conflict of interest.

## Supporting information

Supporting Information

## Data Availability

The data that support the findings of this study are available from the corresponding author upon reasonable request.
