# Supplementary material for: Boosting the Oxidative Potential of Polyethylene Glycol‐Based Polymer Electrolyte to 4.36 V by Spatially Restricting Hydroxyl Groups for High‐Voltage Flexible Lithium‐Ion Battery Applications
Source: Adv Sci (Weinh). 2021 Jun 10;8(16):2100736. doi: 10.1002/advs.202100736 (PMC8373090; doi:10.1002/advs.202100736)
Supplement: Supplementary file 1 — Supporting Information [file ADVS-8-2100736-s001.pdf]

© 2021 Wiley-VCH GmbH

ADVANCED SCIENCE
Open Access

Supporting Information

for *Adv. Sci.,* DOI: 10.1002/advs.202100736

Boosting the Oxidative Potential of Polyethylene Glycol-Based Polymer Electrolytes to 4.36 V by Spatially Restricting Hydroxyl Groups for High-Voltage Flexible Lithium-Ion Battery Applications

*Zhenhan Fang, Yufeng Luo, Haitao Liu, Zixin Hong, Hengcai Wu, Fei Zhao, Peng Liu, Qunqing Li, Shoushan Fan, Wenhui Duan, and Jiaping Wang**

Boosting the Oxidative Potential of Polyethylene Glycol-Based Polymer Electrolytes to 4.36 V by Spatially Restricting Hydroxyl Groups for High-Voltage Flexible Lithium-Ion Battery Applications

*Zhenhan Fang, Yufeng Luo, Haitao Liu, Zixin Hong, Hengcai Wu, Fei Zhao, Peng Liu, Qunqing Li, Shoushan Fan, Wenhui Duan, and Jiaping Wang**

Z. Fang, Dr. Y. Luo, Z. Hong, H. Wu, F. Zhao, Dr. P. Liu, Prof. S. Fan,
Department of Physics and Tsinghua-Foxconn Nanotechnology Research Center, Tsinghua University, Beijing 100084, China

Dr. H. Liu
Laboratory of Computational Physics, Institute of Applied Physics and Computational Mathematics, Beijing 100088, China

Prof. W. Duan
State Key Laboratory of Low-Dimensional Quantum Physics, Department of Physics, Tsinghua University, Beijing 100084, China
Institute for Advanced Study, Tsinghua University, Beijing 100084, China
Frontier Science Center for Quantum Information, Beijing 100084, China

Prof. Q. Li, Prof. J. Wang
Department of Physics and Tsinghua-Foxconn Nanotechnology Research Center, Tsinghua University, Beijing 100084, China
Frontier Science Center for Quantum Information, Beijing 100084, China
E-mail: jpwang@tsinghua.edu.cn

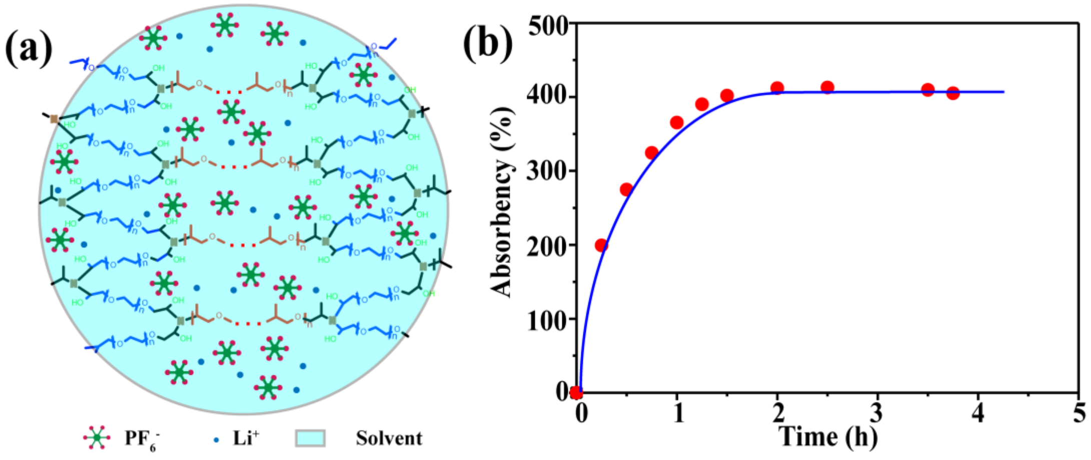

Figure S1 (a) Structural diagram and (b) absorbency curve of the c-PEGR gel.

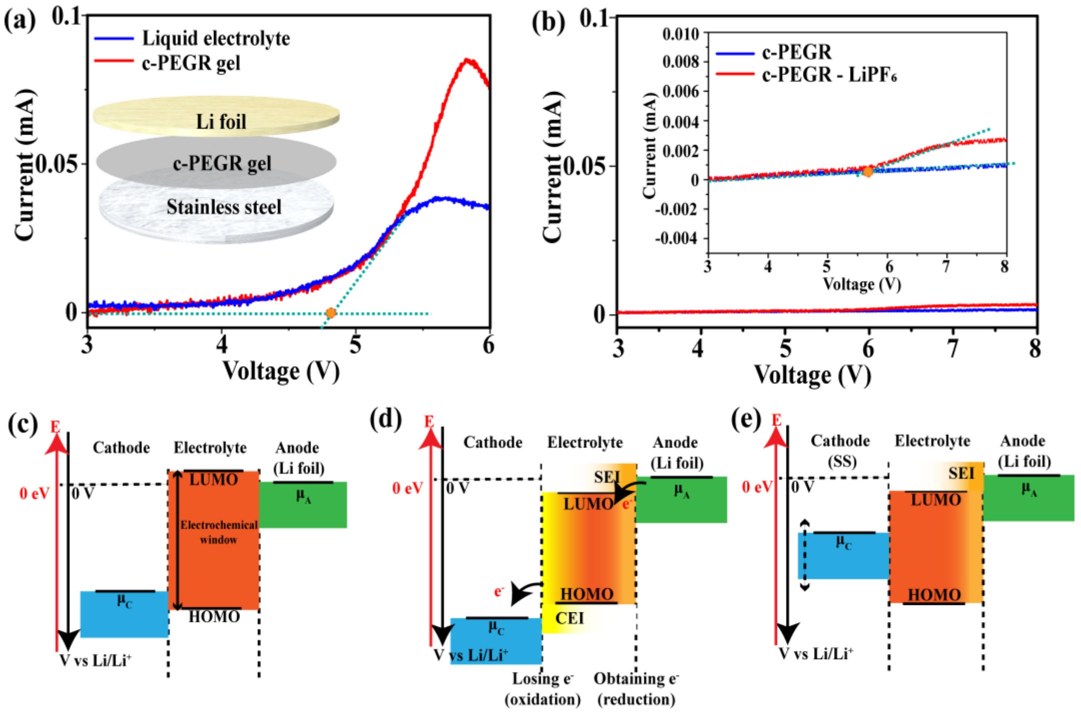

Figure S2 (a) Schematic diagram of cell assembly for oxidation potential measurement (inset) and current-voltage curves of cells with the liquid electrolyte and c-PEGR gel. (b) Current-voltage curves of cells with the c-PEGR and c-PEGR-LiPF6. Diagram of frontline orbital energy levels under (c) ideal condition, (d) adverse condition, and (e) voltammetric measurement.

Figure S2c presents a schematic representation of frontline orbits in an ideal Li metal cell, where the highest occupied molecular orbital (HOMO) energy of the electrolyte is below the chemical potential of the cathode and the lowest unoccupied molecular orbital (LUMO) energy of the electrolyte is above the chemical potential of the anode, and no electron transfer takes place at each interface. Figure S2d, on the contrary, shows a schematic representation of each frontline orbit in a Li metal battery under the most adverse condition. Specifically, at the electrolyte/anode interface, since the LUMO energy of the electrolyte is lower than the chemical potential of the anode, the electrolyte in the interface receives electrons from the anode and undergoes a reduction reaction, which generates new substances and forms an SEI layer. The newly formed SEI membrane needs to have a higher LUMO energy than the chemical potential of the anode to prohibit further reaction. A similar reaction occurs at the cathode/electrolyte interface, where the cathode electrolyte interface (CEI) is the product of the oxidation of the electrolyte at the interface and has a lower HOMO energy level lower than the cathode chemical potential. For the actual voltammetric measurement, since the reference and counter electrode was excess Li metal, its chemical potential barely changed. Therefore, altering the voltage in voltammetry actually adjusted the chemical potential of the cathode by varying the bias voltage.

In addition, since the working electrode was made of stainless steel that could not transfer Li ions, only when the chemical potential of the cathode decreased below the HOMO energy of the electrolyte or increased above the LUMO energy of the electrolyte, electron transfer could occur in the system, so that the current was not expressed as zero.

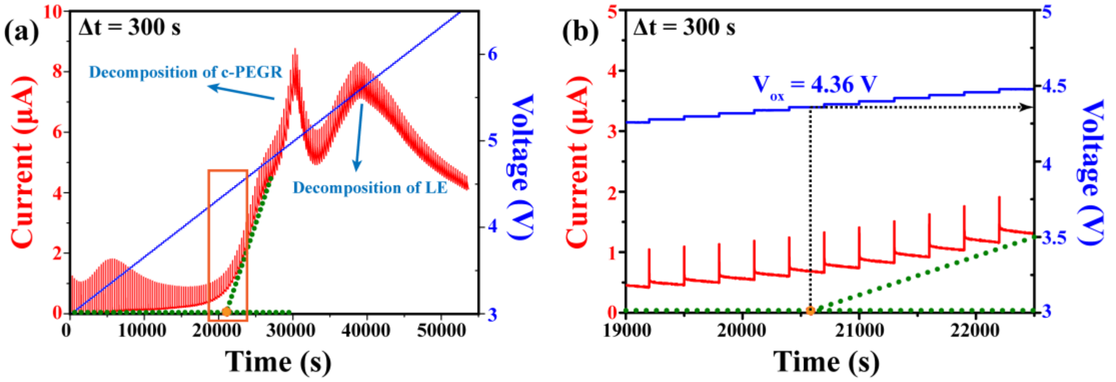

Figure S3 (a) The oxidation potential measurement of the c-PEGR gel using the QS-LSV method at Δt of 300 s and (b) amplified plot.

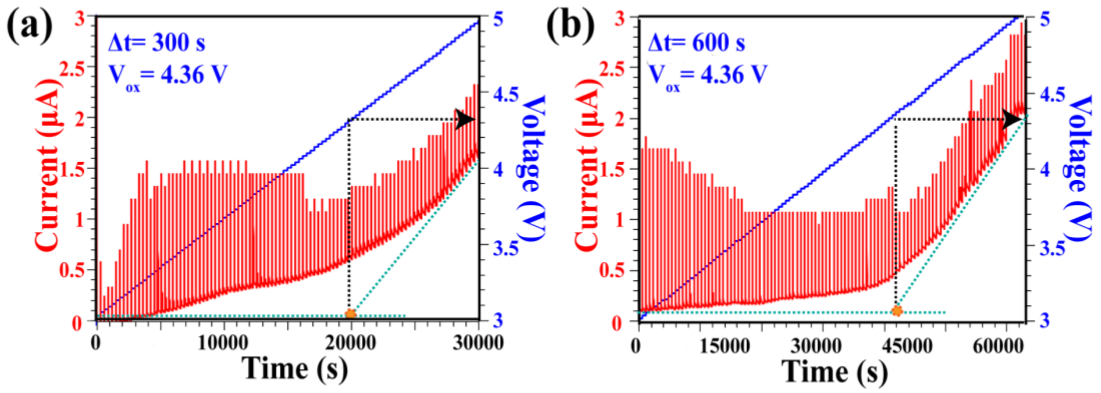

Figure S4 Oxidation potential of the c-PEGR gel measured by setting the QS-LSV at Δt of : (a) 300 s and (b) 600 s.

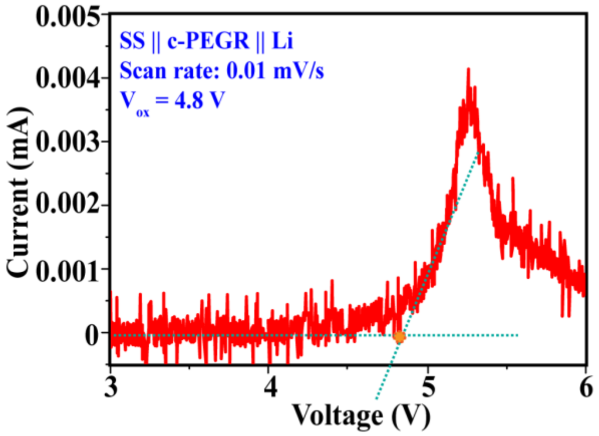

Figure S5 Oxidation potential profile of the c-PEGR gel measured by the conventional LSV method at a scan rate of 0.01 mV s^{-1}.

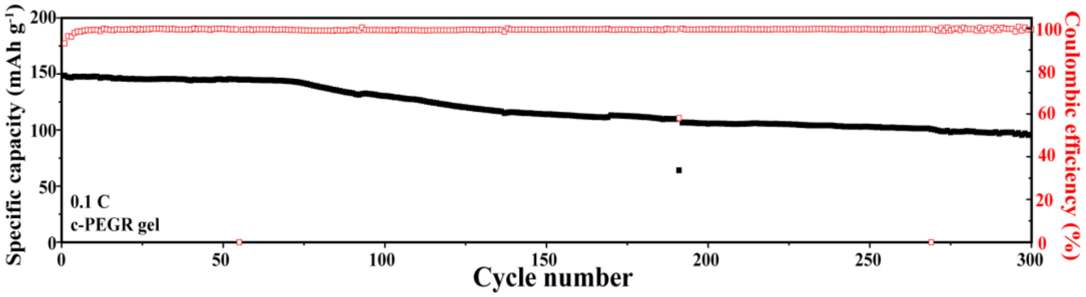

Figure S6 Long-term cycling performance of an LCO||Li cell with the c-PEGR gel at 0.1 C.

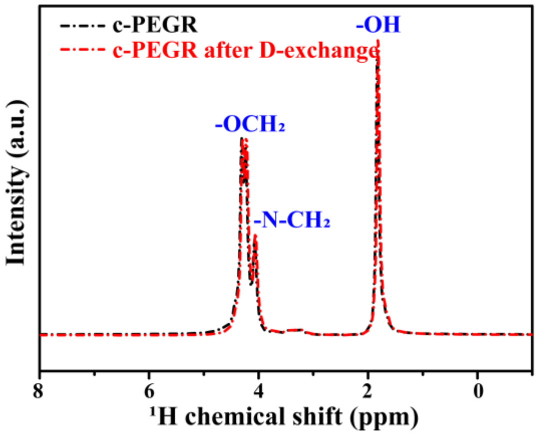

Figure S7 1H-SSNMR spectra of c-PEGR before and after the D-exchange.
